# Relationship between Range Use and Fearfulness in Free-Range Hens from Different Rearing Enrichments

**DOI:** 10.3390/ani11020300

**Published:** 2021-01-25

**Authors:** Md Saiful Bari, Simon S. Allen, Jarrod Mesken, Andrew M. Cohen-Barnhouse, Dana L. M. Campbell

**Affiliations:** 1 School of Environmental and Rural Science, University of New England, Armidale, NSW 2351, Australia; mbari2@myune.edu.au (M.S.B.); jmesken@myune.edu.au (J.M.); Andrew.Cohen-Barnhouse@apvma.gov.au (A.M.C.-B.); 2 Agriculture and Food, Commonwealth Scientific and Industrial Research Organisation (CSIRO), Armidale, NSW 2350, Australia; 3 Department of Dairy and Poultry Science, Chattogram Veterinary and Animal Sciences University, Khulshi, Chattogram 4225, Bangladesh; 4 Agriculture and Food, Commonwealth Scientific and Industrial Research Organisation (CSIRO), Sandy Bay, TAS 7005, Australia; simon.allen@csiro.au

**Keywords:** tonic immobility, open field test, emergence test, vocalisation, behaviour, computer vision, spectrogram, acoustic, chicken, plumage

## Abstract

**Simple Summary:**

In Australia, free-range pullets are reared inside, but the adult hens can go outside. This discrepancy between environments might increase the fearful behaviour of adult free-range hens. Enriching the indoor rearing environments might help reduce this fearfulness. Adult hen fearfulness might also be linked with individual variation in range use. In this study, across 16 weeks of indoor rearing, different types of enrichments were provided to 1700 pullets: weekly changing novel objects, custom-designed perching/navigation structures, or no additional enrichments. Adult hens’ range use was tracked via radio-frequency identification (RFID) systems. A sample of hens (*n* = 135) across all rearing enrichment groups that ranged daily or did not range at all were selected, and several behavioural tests were performed at 62 weeks of age. Overall, the rearing treatments had few effects on the fearfulness behaviour of hens late in the production cycle, and the outdoor rangers were less fearful. Hens that showed higher fear also had poorer feather coverage. Enrichments during rearing showed limited fearfulness reduction for adult free-range hens, and individual variations in range use might be an indicator of bird fearfulness.

**Abstract:**

Inconsistency between the environments of indoor pullet rearing and adult outdoor housing may increase the fearfulness in free-range hens. Rearing enrichments and/or range use may reduce adult fearfulness. Hy-Line Brown^®^ chicks (*n* = 1700) were reared inside across 16 weeks with three enrichment treatments: weekly changing novel objects, custom-designed perching/navigation structures, or no additional enrichments. Pullets were transferred to a free-range system at 16 weeks of age, with range access provided from 25 weeks. At 62 weeks, 135 hens were selected from the three rearing treatments and two ranging groups (indoor: no ranging and outdoor: daily ranging) based on individual radio-frequency identification tracking. Individual behavioural tests of tonic immobility, emergence, open field, and novel object (pen level) were carried out on hens. Spectrograms of vocalisations were analysed for the open field test, as well as computer vision tracking of hen locomotion. The results showed few effects of rearing treatments, with outdoor rangers less fearful than indoor hens. The latency to step in the open field test negatively correlated with hen feather coverage. These results show that individual variation in ranging behaviours is present even following rearing enrichment treatments, and subsequent range use might be an indicator of bird fearfulness.

## 1. Introduction

An animal’s affective state is one major component of animal welfare assessment where individuals can experience both positive and negative affect [1,2]. Fearfulness is a negative state that has evolved as a mechanism for appropriately responding to danger [3], and in welfare assessment, fearfulness can reveal the way an animal copes with its environment [3]. In a threatening situation, the physiological response of birds is activated, which leads to corticosterone release, and thus, fear is also coupled with stress [4]. While an appropriate response to a known or unknown threat is evolutionarily beneficial, chronic and/or excessive fear and anxiety in domesticated poultry may result in poorer welfare [5,6,7]. Management methods to reduce fear responses in laying hens are important for optimising their welfare.

Measuring fear in laying hens is typically done via behavioural tests conducted on the individual or group where separate tests may assess different aspects of a fearful response. A tonic immobility test, for example, is categorised as an antipredator response strategy [8,9]. A novel object test measures exploration and boldness in response to novelty, and an emergence test measures boldness in response to an exposed environment [10,11]. An open field test (OFT) is believed to measure the trade-off between the desire to avoid predator detection (i.e., freezing) and desire for social reinstatement with conspecifics (i.e., vocalisations) [12,13]. Typically, latencies to exhibit specific behaviours are recorded across these tests [5,14], with some researchers observing more detailed behavioural repertoires of the birds during the test period to detect differences among treatment groups [15,16,17]. Vocalisations, however, in open field (or novel arena) tests are most often counted together, either as individual counts of sounds made [18] or as counts of vocalisation bouts, with a limited analysis of the type of vocalisation emitted [17,19]. Laying hens produce different types of vocalisations that may communicate different states of the hen [15,20], and thus, a more detailed acoustic analysis via spectrograms of the vocalisation types made during behavioural tests could provide further insight beyond vocalisation counts. Furthermore, the advent of computer vision technology may enable the automated detection and classification of behaviour greater than that of a human observer. Rozempolska-Rucinska et al. [21] used computer tracker software to analyse the behavioural reactivity of hens based on the head and tail movements in the presence of novel objects and classified shy/bold hens. Kolakshyapati et al. [22] tracked hens’ movements in a novel arena using ANYmaze software, which automatically classified locomotion around the arena. Thus, automated computer-vision movement tracking might reveal behavioural differences during tests beyond what is detected during manual live or video observations.

An animal’s fear response might be influenced by several factors, including their genetics, surrounding social environment, and early life experiences [23]. Research specifically with poultry has shown the rearing period is crucial in preparing hens to cope with later experiences as adults [24]. Several studies have demonstrated reduced fearfulness in hens exposed to increased environmental complexity during rearing [25,26,27,28], including reduced fear when reared with perches in broiler breeders [29]. These early life experiences may act via desensitising the pullets to variable stimuli, increasing the exploration of their environments or improving their capacity to adapt and respond appropriately to stress-inducing environmental changes [30,31]. Free-range hens may particularly benefit from modifications of their rearing environments, as pullets are reared indoors but adult hens have outdoor access. Thus, providing enrichment to pullets during the rearing period may affect the fear and exploration responses of the adult ranging hens.

Free-range hens are further valuable to study, as such wide individual variation exists in the range use patterns of the adult birds. Some hens range outside daily, but others remain inside [14,32,33]. This variation may be associated with fearfulness, as indicated by previous tests demonstrating a higher fearfulness in hens that range less or remain inside [5,14,33], although not all tests conducted both within and among the different studies detected differences [5,14,32,33]. The outdoor environment is more unpredictable and uncontrolled, along with increased predation risks. Thus, it is unclear if the relationship between fearfulness and range use is a causal or consequential effect.

Fearfulness may also be related to other behaviours in laying hens and associated welfare outcomes, such as feather pecking and plumage damage. Plumage losses in laying hens typically result from feather pecking by flock mates, and this is a major welfare problem in laying hen production. The development of feather pecking behaviour and its prevention is multifactorial, with much research into understanding the predictors of its occurrence [34]. Reports on hens from the same flock as the current study, showed hens exposed to rearing enrichments had better plumage coverage than control hens [35,36], but it is unclear if there is also an association with fear. The relationship between fear and feather pecking is not straightforward, as some studies report that increased fear increases feather pecking whereas other research demonstrates increased feather pecking results in greater fear (reviewed in [37]). Measures within behavioural tests have shown correlations with feather pecking behaviour or feather damage (e.g., [38,39]) but not consistently within all studies (e.g., [40]).

Thus, the current study aimed to conduct several behaviour tests on hens from different rearing enrichments and different ranging groups, along with an assessment of their plumage condition. A spectrographic analysis and automated computer vision tracking of the vocalisations and locomotive behaviours, respectively, in the open field test were predicted to reveal group differences beyond what the live counts and video-based observations found. The enriched hens and the outdoor rangers were predicted to be less fearful, with poorer plumage coverage in the more fearful birds.

## 2. Materials and Methods

### 2.1. Statement of Animal Ethics

This experiment was approved by the Animal Ethics Committee (AEC17-092) of the University of New England, Armidale, NSW, Australia.

### 2.2. Pullet Rearing

We reared 1386 Hy-Line^®^ Brown chicks until 16 weeks of age in the Rob Cumming Poultry Innovation Centre (University of New England, Armidale, NSW, Australia), and the adult hens were housed across the laying cycle until 65 weeks of age in the Laureldale free-range facility also located at the University of New England. The rearing and layer housing systems and protocols have been previously described in other datasets from the same larger study [35,36,41]. The pullets were reared indoors in 9 pens (6.2 m L × 3.2 m W) visually separated by shade cloth across 3 separate rooms for up to 16 weeks, with 3 different rearing treatments applied (3 pen replicates per treatment balanced across the different rooms). The rearing treatments comprised a “control” group with standard floor litter (rice hulls) housing only; a “novelty” group where different novels objects such as balls, brooms, brushes, buckets, plastic pipes, strings, containers, bottles, bricks, and pet toys were added and changed at weekly intervals; and a “structural” group where 5 custom-designed H-shaped perching and navigation structures (L, W, H = 0.60 m) with two solid panels and one open-framed side were placed in different orientations within the pens. These structures provided approximately 7 cm of elevated perching space per bird, with an additional area for the birds to stand on, stand next to, or go under, as the solid panels and flexible orientations were incorporated to encourage navigation within the pen, in addition to perching. Commercially formulated mash in round feeders and nipple drinkers were provided for ad libitum feed and water access as per the current Australian Model Code of Practice for the Welfare of Animals—Domestic Poultry [42]. Lighting and temperature schedules were maintained as per the Hy-Line^®^ Brown alternative management recommendations [43], but artificial LED lighting was maintained at 100 lux as the pullets were destined for outdoor access. The bird density was approximately 15 kg/m^2^ at 16 weeks of age. Due to delivery error, 1700 chicks were reared in total, but only 1386 were transferred to the layer facility with, first, lighter, heavier, and then some randomly selected extra pullets rehomed at 16 weeks. No cooling system was available, but the pens were fan-ventilated and heated when required. The chicks were beak-trimmed at the hatchery and vaccinated as per the standard recommendations.

### 2.3. Free-Range Housing

The pullets were remixed among replicates within the same treatments when transferred at 16 weeks of age and housed within 9 pens again (3 replicates/treatment) within a single shed. Each of the 9 pens included 2 small nest boxes, a two-tiered large nest box providing 133 cm^2^ nest space/hen, 2 large, round hanging feeders, and a series of water nipples and perches (10 cm space/bird due to pen space limitations, but birds also perched on the waterlines and feeder rims), as per the requirements of the Australian Model Code of Practice for the Welfare of Animals—Domestic Poultry [42], and were visually isolated via shade cloth on the wire partitions. Drinking water and commercially formulated food were provided ad libitum. Rice hulls were placed as a deep litter substrate, with one complete litter replacement at the mid-period of the laying cycle. The LED lighting schedule gradually increased to 16-h light and 8-h dark by 30 weeks of age, with an average pen intensity of 10.0 (±0.84 SE) lux (Lutron Light Meter, LX-112850; Lutron Electronic Enterprise CO., Ltd., Taipei, Taiwan) as measured at the birds’ eye height from 3 pen locations (front, middle, and back) when the pop-holes were closed. The shed was fan-ventilated only, with no additional environmental control. Each pen was connected to an outdoor range (31 m L × 3.6 m W) through 2 pop-hole openings (18 cm W × 36 cm H). The range area from the pop-holes onwards was 1.1 m of concrete path followed by 1.6 m of river rocks, then a grassed area with no shelter or trees. The amount of forages in the range varied greatly due to hen access and seasonal variations. The range area was separated visually for each pen via a shade cloth. The hens were first given outdoor access at the age of 25 weeks (May 2018), and the pop-holes automatically opened at 9:15 a.m. and closed after sunset daily henceforth. Thus, the hens were provided with approximately 9 h of range access daily in the winter and 11 h in the summer following daylight saving time (October 2018).

### 2.4. Radio Frequency Identification (RFID) Systems

All hens were fitted with adjustable leg bands (Roxan Developments Ltd., Selkirk, Scotland) containing glued microchips (Trovan^®^ Unique ID 100 (FDX-A): operating frequency 128 kHz, Microchips Australia Pty Ltd., Keysborough, VIC, Australia) to track their daily movement in and out of the range pop-holes via radio-frequency identification (RFID) systems. The RFID systems were designed and supported by Microchips Australia Pty Ltd. (Keysborough, VIC, Australia), with the equipment developed and manufactured by Dorset Identification B.V. (Aalten, The Netherlands) using Trovan^®^ technology (RFID Systems Ltd., North Ferriby, United Kingdom). The RFID system recorded the date, time, and direction (onto the range or into the pen) of each tagged bird passing through with a precision of 0.024 s (maximum detection velocity 9.3 m/s).

### 2.5. Selection of Hens

RFID data of all hens were collected from 56 to 62 weeks of age (45 days) and were first run through a custom-designed software program written in the “Delphi” language (Bryce Little, CSIRO, Agriculture and Food, St Lucia, QLD, Australia [44]) to exclude any “false” unpaired readings that may occur when a hen does not complete a full movement transition between the pen and range. The program also summarised the daily time spent outside for each hen and the percentage of available days that a hen accessed the range. From these data, a total of 135 hens with different ranging patterns were selected from 9 pens across the three rearing treatments. The two ranging groups were defined as “indoor”, which were hens who did not range at all, and “outdoor”, for hens that ranged daily and for the longest period. Hens that were tested in similar behavioural tests as part of separate datasets [5] were not included. At least 20 “indoor” and 20 “outdoor” birds were selected from each rearing treatment group across all the pens. A total of 44 hens from the control group of rearing treatments (20 indoor and 24 outdoor), 45 hens from the novelty group (22 indoor and 23 outdoor), and 46 hens from the structural group (24 indoor and 22 outdoor) were selected across all the pens.

All the selected hens were banded with additional leg bands to detect them easily in their home pens during testing days. A combination of extra leg bands was removed or added after each behaviour test to identify tested and untested birds. All the behavioural tests were conducted by a single trained experimenter who was blinded to the rearing and ranging treatments, with tests video-recorded for further observations. An unequal number of hens across rearing treatments and ranging patterns were selected and tested, as it was anticipated that some hens may be unable to be found within the pen of 154 hens (based on past experiences), but all selected hens were able to be tested, and thus, their data were included to avoid biased data exclusion.

### 2.6. Behavioural Tests

All testing occurred from 62 weeks until 63 weeks of age from approximately 9:00 a.m. until 5:00 p.m. each day in a room adjacent to the main housing shed. All individual tests were applied in the same order (as listed in the following sections) for each hen, with at least 3 days between different tests for each hen. Individual hens were randomly selected from the available banded hens within each pen, with the daily testing order balanced across pens and rearing treatments. All hens were returned to their home pens immediately following the completion of their test. The pop-holes were not closed during individual testing times, but ranging hens were able to be captured when they periodically returned inside.

#### 2.6.1. Tonic Immobility (TI) Test

Hens were captured inside their home pen and moved to the adjacent room. The tonic immobility test was performed by placing the bird in a flat position on their back in a metal frame with their head hanging down at one end. The experimenter’s right hand gently held the breast of the bird, and the left hand gently held the bird’s head down. Hens were kept restrained for 5 s in this position; then, the experimenter removed their hands and stepped to one side with their eyes averted downwards. The duration the bird remained immobile was recorded up to a maximum of 5 min (300 s). However, if the bird righted themselves within 10 s, another attempt was applied, up to a maximum of 5 attempts, until tonic immobility was induced for a minimum of 10 s.

#### 2.6.2. Open Field Test (OFT)

Individual birds were placed into a wooden open field arena (1.8 m × 1.8 m) in the adjacent testing room with the lights turned off. The arena had a shade cloth ceiling to minimise escape attempts with an opening in the centre to allow video recording. A small opening was at the bottom of the back of the box to allow bird placement. Two cameras (Sony Handycams HDR-PJ410, Sony Corporation, Tokyo, Japan) were placed at the top to both record the hens and to observe the bird in real-time on a connected monitor. After placing the hen, the lights were switched on, and the test commenced. A trained observer recorded bird behaviour via the connected video screen within the same room but out of sight of the hen. The latency to first move (more than just the head but without taking a step), latency to step, latency to vocalise, and the number of vocalisations (each sound a bird made) per minute were recorded live across the 5-min test.

**Figure 1:**
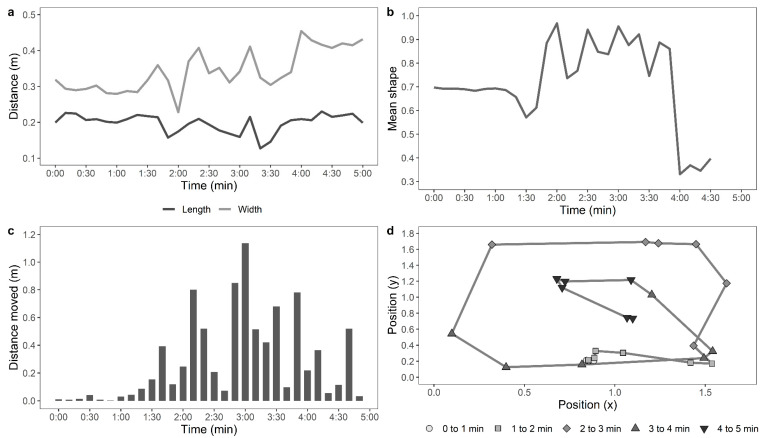
Automated computer tracking to show: (**a**) distance moved across time by a relatively active hen along the length and width of the test arena, (**b**) mean shape of the hen based on a superimposed ellipse, where a value of 1 is a full circle, and a value of 0.5 indicates a shape twice as long as wide, (**c**) distance moved across the test duration, and (**d**) position of the moving hen across time (parameters are described in greater detail in Table 1).

**Figure 2:**
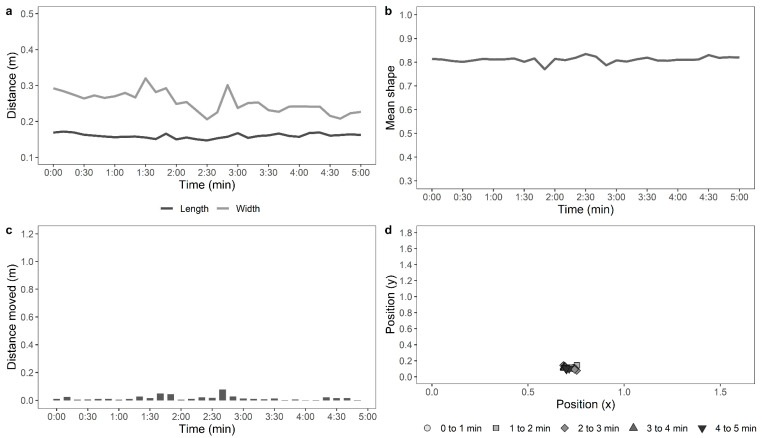
Automated computer tracking to show: (**a**) distance moved across time by a relatively stationary hen along the length and width of the test arena, (**b**) mean shape of the hen based on a superimposed ellipse, where a value of 1 is a full circle, and a value of 0.5 indicates a shape twice as long as wide, (**c**) distance moved across the test duration, and (**d**) position of the stationary hen across time (parameters are described in greater detail in Table 1).

Later, a single trained experimenter who was blind to the ranging and rearing treatment groups observed the videos to count the number of steps made by each hen per minute in the test arena. The distance travelled in the arena by each of the hens during the testing time was also assessed from the recorded videos automatically using custom-developed movement tracking software by personnel blinded to the rearing and ranging treatment groups. Each image file was opened in python using a combination of OpenCV (https://pypi.org/project/opencv-python/) and the simple object tracker Tracktor (https://github.com/vivekhsridhar/tracktor). Each frame was reduced to ¼ of its original size and passed through the Tracktor object detection algorithm. An ellipse was fitted to the resulting “blob”, which represented the chicken’s shape. For each frame, the centre of mass of the blob was recorded and the centre of the fitted ellipse, along with the length of the ellipse’s major and minor axes and the ellipse orientation. For a five-minute file with 100% detection, this generated (25 × 60 × 5) 7500 records. The full record was split into 10-s windows for measuring the parameters, as described in Table 1. Examples of the movements of an active, moving hen and a stationery hen are shown in Figure 1 and Figure 2, respectively.

The vocalisations of the hens were also recorded using a Marantz Professional Solid-State recorder PMD661MKII (Marantz Professional, Cumberland, RI, USA) and a Sennheiser ME67 Condenser microphone (Sennheiser, Wedemark, Germany) at a sampling rate of 48 kHz and 24-bit accuracy. The microphone was installed at the top of the test arena and recorded all vocalisations for the duration of the test. Spectrograms of the vocalisations were evaluated using audio analysis software (Raven Pro 1.6, Cornell Laboratory of Ornithology, Cornell University, Ithaca, NY, USA; www.birds.cornell.edu/raven); 1792-sample Hann window, 38.5-Hz filter bandwidth, 5.86-Hz frequency resolution (grid spacing), discrete Fourier-transform (DFT) size of 8192 samples, and a time grid hop size of 200 samples (88.8% overlap)), with the spectrogram parameters matching (where possible) those reported by McGrath et al. [15]. All vocalisations were viewed by the same experimenter displayed on the same screen. For each hen, the number of calls produced across the 5-min test and the total number of syllables (individual sounds) per call were counted from the spectrograms. Syllables with a time lapse of >25 ms in between were classified as separate calls. Call types based on McGrath et al. [15] were unable to be clearly distinguished, and many calls were observed to be “mixed calls” (comprised of different types of syllables), which were excluded from the analysis in McGrath et al. [15]. To avoid the exclusion of a large portion of the vocalisations, the parameters of all individual syllables produced for each hen were analysed and included in the data, as detailed in Table 2. All measurements were conducted on the fundamental frequency for each syllable. Individual syllables that were not able to be distinguished clearly enough on the spectrogram were not analysed. A total of 9 hens produced 27 unclear syllables (control: *n* = 6 syllables, novelty: *n* = 16, structural: *n* = 5, indoor: *n* = 17, and outdoor: *n* = 10).

#### 2.6.3. Emergence Test (ET)

Individual birds were placed into an enclosed wooden emergence box (60 cm L, W, and H) with a sliding door at the front that was located inside a mesh dog cage (3 m × 2.2 m) within the main housing shed to provide a similar environment to the pens in which they were housed. Hens were familiar with the dog cage, as it was used during regular weighing and welfare scoring sessions on all the hens [35], but a bright light was set to make it a novel environment for the birds, simulating the transition out of a pop-hole. Two Arlec 500-W halogen work lights (Arlec, Blackburn North, VIC, Australia) were set on both sides at the top of the box shining downwards at the box entryway with a combined intensity of 3100 lux (Lutron Light Meter, LX-112850; Lutron Electronic Enterprise CO., Ltd., Taipei, Taiwan) measured at the hens’ eye level close to the entrance of the emergence box. A shade cloth was hung partway down over the entrance to minimise light going directly into the box but to still allow the hens to freely walk out. Hens were captured from their home pens, placed in the emergence box, and the door was closed. Thirty seconds following placement, the door was slid open with the bright lights turned on. An observer out of sight of the bird recorded the latencies to first step out of the box (one leg past the door frame) and to completely emerge (whole body past the door frame) up to a maximum test time of 5 min.

#### 2.6.4. Novel Object Test (NOT)

Novel object tests were carried out on the groups of birds within their pens prior to the pop-holes opening in the morning. All pens were tested sequentially across a period of approximately 2 h across 2 days. A novel object was placed consecutively in 3 locations within the home pen, including the front-centre (next to the door), mid-centre, and centre-back (near the pop-holes). After placing the object, the trained experimenter (located within the pen) who was blinded to the rearing treatments observed the hens’ behaviours up to 5 min. The hens were accustomed to people in the pens from daily husbandry procedures, and the hen strain used did not avoid people as adult birds. The latencies for the first 3 birds to peck the object at each site within the pen were recorded. This was performed across all pens with 2 different novel objects, including a cardboard cereal box and a shiny red ball, across 2 separate days. These exact objects were not among those provided to the novelty group during the rearing period.

#### 2.6.5. Plumage Coverage of Tested Hens

At the completion of all behavioural tests at 63 weeks of age, the plumage condition of all tested hens were assessed by a single experimenter blinded to the rearing and ranging treatments using the scoring system described by Tauson et al. [46]. In this scoring system, 4 scores were available for feather coverage, with a score of 4 indicating the maximum feather coverage, whereas a score of 1 indicated no plumage, just bare skin. The front of the neck was scored separately from the back of the neck and was not included in the analyses, as the majority of damage on the neck front was likely due to rubbing on the feeder rims whilst feeding rather than as a result of pecking damage. A maximum score of 24 could be obtained for the feather conditions across 6 body parts, including the neck, chest, back, wing, vent, and tail.

### 2.7. Data and Statistical Analyses

All data were analysed using JMP^®^ 14.0 (SAS Institute, Cary, NC, USA), with α set at 0.05. Where applicable, the nonsignificant interactions were removed from the final model. Where significant differences were present, post-hoc Student’s *t*-tests were performed. Bonferroni corrections were applied for more than 3 post-hoc comparisons. Data were transformed for analyses where necessary, but the raw values are presented in the figures and tables.

The individual bird latencies measured during the tonic immobility test (TI) (*n* = 135), open field test (OFT) (*n* = 135), and emergence test (ET) (*n* = 135) were compiled. For the duration of TI, latencies to move, vocalise, and step in the OFT, and latencies to step out and emerge in the ET, general linear mixed models (GLMMs) were performed after a log_10_ transformation of the latencies. In the GLMMs for the individual behavioural tests, the rearing treatments and ranging, along with their interaction, were fixed effects, and IDs of birds nested within pen, and pen nested within rearing treatment, and ranging were random effects.

The count data per individual bird for the number of vocalisations and the number of steps in the OFT were square root-transformed. GLMMs were applied, with rearing treatment, ranging, and time (each minute of the test), along with their interactions, as fixed effects, and IDs of birds nested within pen, and pen nested within rearing treatment and ranging as random effects. From the audio recordings, the total number of calls and the number of syllables per call produced by each hen were compiled and square root-transformed. GLMMs were fitted, with rearing treatments, ranging, and their interactions as fixed effects and IDs of birds nested within pen, and pen nested within rearing treatment and ranging as random effects. A total of 1159 individual syllables were analysed via spectrograms across all hens. The variables of frequency 75%, interquartile frequency, frequency 95%, bandwidth 90%, delta frequency, and delta time from the individual syllables were log_10_ transformed. GLMMs were then applied, with rearing treatment, ranging, and their interaction as fixed effects and with IDs of birds nested within pen, and pen nested within rearing treatment and ranging as random effects.

For the automated tracking of hens in the OFT, the data for the distance moved by the hens, mean shape of the hens, standard deviation from the mean shape, maximum 10-s move by the hens, stationery time of hens, and the move ratio were compiled (*n* = 117). The data from 18 hens (control: *n* = 5, novelty: *n* = 7, structural: *n* = 6, indoor: *n* = 11, and outdoor: *n* = 7) were removed, as the automated recognition detected less than 75% of the hen’s movements due to difficulties in distinguishing paler-coloured hens from the wooden floor of the arena. The distance moved by the hens and maximum 10-s move data were log_10_ transformed, and the move ratio was logit transformed. GLMMs were fitted separately for each parameter, with rearing treatment, ranging, and their interaction as fixed effects and with IDs of birds nested within pen, and pen nested within rearing treatment and ranging as random effects. The live counted “number of steps” was square root-transformed, the automated “distance travelled (m)” by the hens was log_10_-transformed, and a Pearson’s correlation analysis was applied to them.

The latencies of the group-level novel object tests using the cardboard box and red ball were compiled for each of the 9 pens at 3 different testing locations (*n* = 81/NO: 9 pens × 3 locations × first 3 birds to peck). The latency data were log_10_-transformed and GLMMs were applied. In the GLMMs, the rearing treatment and location of the novel object placements, along with their interaction, were fixed effects, and pen nested with rearing treatment was a random effect. The fixed effect of “bird” was included in the interactions but not in isolation.

The feather score data were square root-transformed, and general linear models were applied to assess their relationship with the log_10_-transformed duration of TI; latencies (s) to first move, step, and vocalise in the OFT; and latencies to step out and emerge in the ET. A Pearson’s correlation was also performed between the log_10_-transformed latencies of the behavioural tests and the square root-transformed feather scores of the hens.

## 3. Results

### 3.1. Behaviour Tests (Individual Level)

#### 3.1.1. Tonic Immobility (TI)

The rearing treatments (*p* = 0.39) and ranging (*p* = 0.26) had no effect on the duration of TI (Table 3), and there was no significant interaction between these variables (*p* = 0.62).

#### 3.1.2. Open Field Test (OFT) Latencies

The rearing treatments did not affect the latency to first move (*p* = 0.41), latency to vocalise (*p* = 0.54), and latency to step (*p* = 0.10) in the OFT. Ranging did have an effect, with the indoor hens taking longer to both first vocalise (*p* = 0.01) and step (*p* = 0.003) than the outdoor hens, but there was no significant difference in the latency to first move (*p* = 0.26; Table 3). There were also no significant interactions between the rearing treatments and ranging for any of these variables (all *p* ≥ 0.37).

#### 3.1.3. OFT Vocalisations and Steps

**Figure 3:**
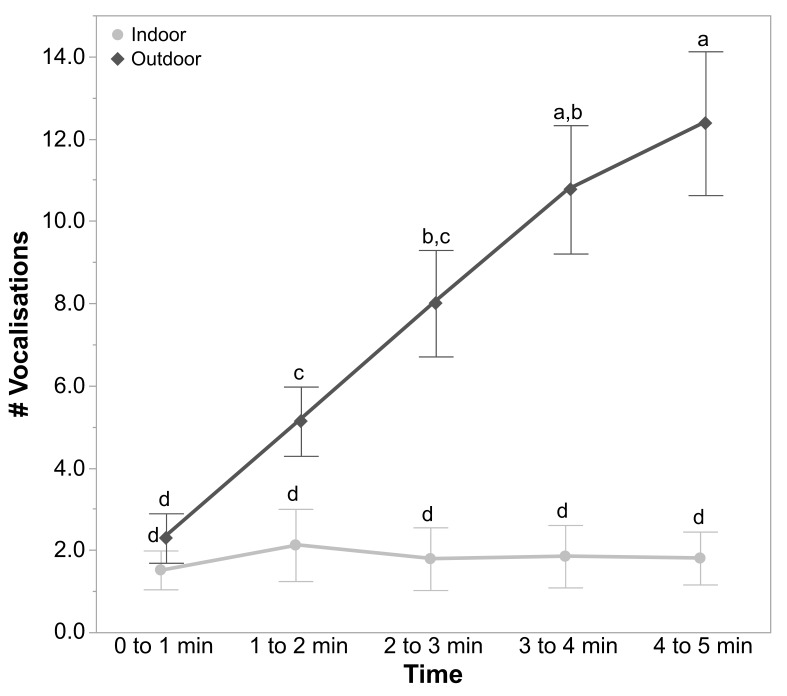
The mean ± SEM of the number of vocalisations produced across time in the open field test (0 to 1 min, 1 to 2 min, 2 to 3 min, 3 to 4 min, and 4 to 5 min) by hens of different ranging patterns (indoor and outdoor). ^a–d^ Dissimilar superscript letters indicate significant differences between the ranging patterns across time (*p* < 0.005). Raw values are presented, with the analyses conducted on the transformed data.

**Figure 4:**
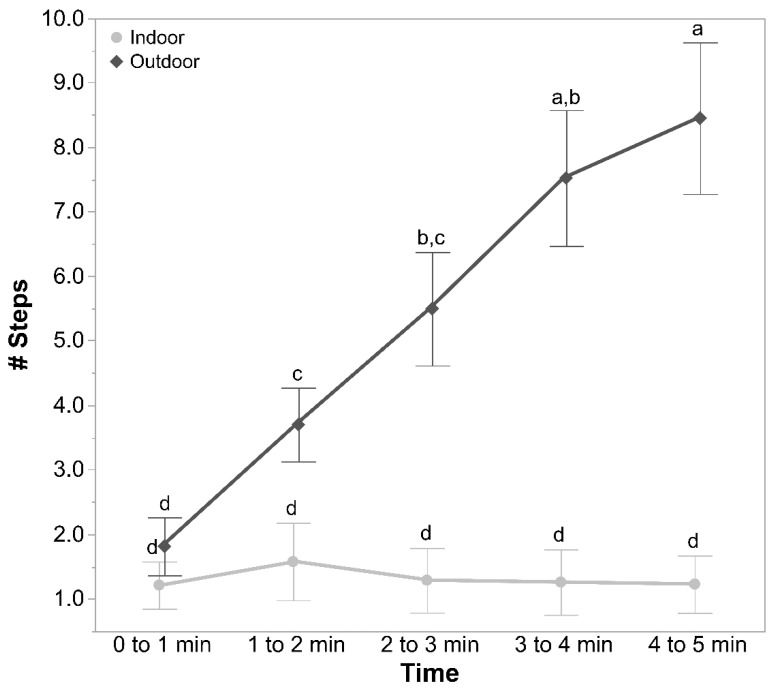
The mean ± SEM of the number of steps made in the open field test across time (0 to 1 min, 1 to 2 min, 2 to 3 min, 3 to 4 min, and 4 to 5 min) by hens of different ranging patterns (indoor and outdoor). ^a–d^ Dissimilar superscript letters indicate significant differences between the ranging patterns across time (*p* < 0.005). Raw values are presented, with the analyses conducted on the transformed data.

The rearing treatments did not affect the number of vocalisations (F_2, 13_._40_ = 2.07, *p* = 0.16) and the number of steps (F_2, 13_._44_ = 1.71, *p* = 0.22) in the OFT. However, there was a significant interaction between the ranging patterns of hens and time for the number of vocalisations (F_4, 532_ = 18.81, *p* < 0.0001; Figure 3) and the number of steps made (F_4, 532_ = 16.70, *p* < 0.0001; Figure 4) by the hens. The outdoor hens made increasingly more vocalisations and steps across time than the indoor hens (Figure 3 and Figure 4). There were no other significant interactions for any of the measured parameters (all *p* ≥ 0.59).

#### 3.1.4. Automated Tracking of Hen Movement in the OFT

The total distance moved by the hens in the automated tracking and the number of steps by the hens in the live counts in the OFT were strongly positively correlated (Pearson’s *r* = 0.95, *p* < 0.0001). The rearing treatments affected the distance moved by the hens (*p* = 0.04), with the novelty hens travelling a greater distance than the control and structural hens (Table 4). The mean shape of the novelty hens was smaller than the control, but not the structural, hens (*p* = 0.04). The novelty hens stayed stationery for a shorter period of time than the control and structural hens (*p* = 0.03, Table 4). The outdoor hens moved a greater distance (*p* = 0.0006) in the OFT arena and stayed stationery for a shorter period of time (*p* = 0.001), with higher maximum moves in 10 s (*p* = 0.0001) and higher move ratios (*p* = 0.008) than the indoor hens. The standard deviation of the shape of the outdoor hens was higher than the indoor hens (*p* = 0.0001; Table 4). There were no significant interactions between the rearing treatment and ranging for any of the measured parameters (all *p* ≥ 0.14). A GLMM on the summed number of manually counted steps with the same 18 hens excluded (fixed effect of the rearing treatment, IDs of birds nested within pen, and pen nested with rearing treatment and ranging as the random effects) showed a trend for significance (F_2, 7_._83_ = 3.60, *p* = 0.08), where the post-hoc tests showed the novelty hens stepped more than the control hens.

#### 3.1.5. Hens’ Calls and Spectrogram Analyses

The rearing treatments did not affect the number of calls (*p* = 0.63) and the number of syllables per call (*p* = 0.66) produced by the hens in the OFT, but the outdoor hens produced more calls than the indoor hens (*p* = 0.02; Table 5). There was no difference in the number of syllables per call between the indoor and outdoor hens (*p* = 0.10; Table 5). There were no significant interactions between the rearing treatments and ranging for the number of calls and the number of syllables per call (both *p* ≥ 0.72).

The rearing treatments did not affect any of the frequency parameters measured in the syllables (all *p* ≥ 0.19), but the delta time was higher in the syllables produced by the structural hens than the novelty hens (*p* = 0.06; the post-hoc test showed a difference). The outdoor hens vocalised at higher frequencies at the 5% measure in the syllables than the indoor hens (*p* = 0.04; Table 6). There were no significant interactions between the rearing treatment and ranging for any of the measured parameters (all *p* ≥ 0.37).

#### 3.1.6. Emergence Test (ET) Latencies

The rearing treatments did not affect the latency to step out (*p* = 0.92) and the latency to emerge (*p* = 0.92) from the test box, but the indoor hens took longer to both step out (*p* = 0.0004) and emerge (*p* = 0.0001) from the test box than the outdoor hens (Table 3). There were no significant interactions between the rearing treatments and ranging for either of these variables (both *p* ≥ 0.15).

### 3.2. Behavioural Tests (Group Level)

#### Novel Object Test (NOT)

The rearing treatments did not affect the latencies to peck the cardboard box (*p* = 0.24) or the red ball (*p* = 0.27). Hens took the longest time to peck the box in the first location and the least time in the third location (*p* < 0.0001; Table 7), with no significant interactions between the fixed effects (all *p* ≥ 0.14). Hens took longer to peck the red ball in the first and second locations than the third location (*p* = 0.0003), with no significant interactions between the fixed effects (all *p* ≥ 0.55; Table 7).

### 3.3. Latency of Behaviour Tests versus Plumage Coverage Score

The regression analysis showed that, as the latency(s) to step in the OFT increased, the plumage coverage of hens significantly decreased (r = −0.19, R^2^ = 0.04, F_1, 133_ = 5.02, *p* = 0.03). The relationships of the duration of TI, latencies to first move and vocalise in the OFT, and latencies to step out and emerge in the ET were not significant (all *p* ≥ 0.12).

## 4. Discussion

This study assessed the effects of rearing enrichments and range use on the fearfulness of 62 to 63-week-old free-range hens using several behavioural tests, along with the relationship of fearfulness with hen plumage coverage. The rearing treatments had few impacts on the fearfulness measures, but the hens that did not range showed greater fear than the hens that ranged daily. The more fearful hens were in the open field test (OFT), the poorer their plumage condition. A spectrographic analysis of their vocalisation syllables did not significantly distinguish the treatment groups more than the live counts of individual sounds made during the OFT, but the automated tracking of hen movements revealed some differences between the treatment groups better than the manual counts of steps alone.

The rearing treatments did not affect the fearfulness behaviours of hens, as quantified by the observer measures taken in the individual-bird tonic immobility (TI), emergence test (ET), and the OFT. However, the automated tracking of birds in the OFT did reveal some rearing group differences, with the novelty hens moving a greater distance and staying stationary for a shorter period of time than the control and structural hens, suggesting these hens were less fearful, more active, and more explorative [47,48]. These inconsistencies across the tests reflect the different aspects of the behavioural traits that each test assessed. While the tests may be categorised as all measuring fear, the avoidance of a novel object may be different than the avoidance of a novel environment, and bird responses may be context-specific [11]. More detailed measures may be necessary to detect subtle differences that the primary test measures did not assess. The results differentiating the novelty hens were similar to those of Jones and Waddington [49], where chicks reared with novel items and tested individually as chicks walked more in the open field test, as well as approached novel objects quicker. Pullets reared in more complex aviary environments approached novel objects more as young adults compared with cage-reared hens [28], and the provision of perches for broiler breeder pullets reduced the TI durations of young adult birds [29]. Thus, the structural hens may have also been expected to show reduced fear relative to the control hens. In a different subset of hens from the same flock tested in an open field test at 20 weeks of age, the structural hens were the quickest to first step (Campbell et al., unpublished data). Pen-level rearing treatment differences in the novel object test may have been detected with a larger number of pen replicates, which were restricted in this study by the capacity of the research facility. Individual-bird novel object tests were not conducted, as the more fearful birds freeze in a novel test environment, which may negate measuring responses to the novel object specifically. Experiences and environments in early life are crucial in the development of fearfulness and preparing birds to cope with encounters experienced later in life [24,50,51]. However, in this study, the measures of fearfulness in the adult birds later in the lay cycle were related more to range use variations than to their rearing environments, suggesting the influence of experiences during the laying period [52]. However, these birds were only tested late in the laying cycle, and thus, it is unknown whether they would have shown greater impacts from their rearing treatments if they were tested directly following the rearing period or directly prior to their first range access. Behavioural tests on different subsets of birds from the same larger flock indicated some differences in fearfulness detected in the pullets and young adults across the rearing treatments (before the pop-holes first opened) but not consistently among all the applied tests (Campbell et al., unpublished data). While rearing enrichments did affect the ranging behaviours and welfare of the birds across the flock cycle [35,36,53], impacts on the measures of fearfulness tested in this study were minimal.

In contrast with the minimal impacts of rearing enrichments, outdoor rangers were found to be less fearful than the indoor hens across the OFT (both observer and automated measures) and ET, although not TI. Previous studies have also found lower-ranging hens to be more fearful [5,14,22,32,33], but not all tests across all studies reveal differences, with the TI duration showing a negative correlation with the range use in some studies [32,54,55] and no relationship in others [5,14,56]. Behavioural tests on the same hens do not always correlate and show the same differences, which is likely related to different specific aspects of the traits they are measuring. Thus, it is recommended to apply multiple tests to ensure that the potential differences are detected [11]. Hens with greater fear may be less willing to explore the outdoor range where there is open exposure and greater variability in the environmental conditions. The exposure to the range itself may then improve the adaptability of the birds or habituate them to new stimuli and, thus, decrease their fear with the increasing range use. Range use itself increases over time [22,53], and thus, while there may be some effects of underlying behavioural traits on the initial range access (Campbell et al., unpublished data), these traits may be modified by experiences across the lay cycle. However, the direction of the relationship between fearfulness and range use is not clear from this study, and further research applying fearfulness tests both prior to and following range usage may confirm the causality.

In addition to applying multiple tests to the same sets of birds, the measures taken within specific tests may also determine whether significant differences are detected or not. A spectrographic analysis of the vocalisation syllables during the OFT was conducted to determine if this greater detail of analysis would better differentiate the treatment groups. Previous studies have indicated that chicks will produce distress vocalisations, and hens may produce alarm calls in the OFT [13,39,57]. These vocalisations communicate a higher state of arousal than other types of calls, which could be missed by categorising all emitted sounds as the same. A spectrographic analysis of the vocalisation type and syllable structure has distinguished the anticipatory behaviour of hens in response to varying types of rewards [15], and categorising vocalisations as “loud” or “soft” has previously distinguished beak-trimming treatment groups [19]. While we were unable to reliably categorise the call types in this study, the hens were not observed to produce alarm calls, and counting the total sounds produced was able to distinguish between the ranging treatment groups. The detailed spectrographic analysis revealed few other differences between the ranging and rearing treatments in response to the open field environment. The outdoor hens did have a higher average minimum frequency (frequency 5%) than the indoor birds, which could be reflective of the exposure to different acoustic environments daily. The range environment was set in a rural area but close to a road, and the birds would have been exposed to cars/trucks, as well as wild birds. Comparatively, birds inside the shed would have heard mainly other chickens, with few additional noises. Adjustments of the minimum frequencies have been observed in wild birds in urban versus nonurban areas [58], with the relationship between range access and vocalisation structure requiring further investigation. A vocalisation analysis may be valuable for other individual-level tests (e.g., in an attention bias test with an alarm call playback [5]) or other treatment groups, and thus, further studies are still warranted to explore the potential for spectrogram analysis of hen calls during behavioural tests.

The plumage coverage was also related to fearfulness, with hens of better plumage showing a shorter latency to step in the OFT; this shorter latency to step was also associated with a higher range use. Feather loss is predominantly a consequence of feather pecking behaviour. However, only the OFT showed a relationship confirming the multifactorial potential causes and/or consequences of feather pecking. Fearful hens might be less active and explorative and, thus, were able to be pecked more by other hens [59]. These hens might spend less time exploring and foraging outside, thus increasing the time spent indoors, which may increase the probability of being a pecking target. These results are similar to what was found in the overall flock of hens across the study period, where lower-ranging hens had poorer plumage coverage [35]. Range use and plumage coverage may also be affected by the thermal comfort of the birds, with feather-pecked hens preferring to remain inside where the temperatures may be more stable and exposed skin is protected from the sunlight. Previous research has shown that hens observed farther out on the range have better feather coverage than those observed closer to the shed [60]. Thus, there may be a relationship between fear, range use, plumage damage, and thermal comfort that has consequences for the optimal management of hens in free-range production systems.

## 5. Conclusions

Overall, the rearing enrichments had few effects on the fearfulness behaviours of the hens at the later stage of their laying cycle, but outdoor rangers were less fearful than the indoor hens. This confirms previous comparisons between indoor and outdoor free-range hens and highlights the importance of individual behavioural trait variations on the use of the range resource. The variations in the relationships between behavioural tests and plumage damage confirm the need for multiple tests on each bird, as only the open field test in this study showed that more fearful hens had poorer plumage. A spectrographic analysis of the vocalisation syllables did not significantly distinguish the treatment groups more than live counts of individual sounds made during the open field test, but more detailed behavioural analyses such as automated computer tracking could reveal behavioural differences beyond the measures made via video observations.

## Figures and Tables

**Table 1:** Description of the measurements used in the automated tracking of hens in the open field test.

Measurement	Description
Percent detection (%)	The percentage of the file that the tracking algorithm found a chicken; this was more difficult with paler, speckled brown hens compared with the more consistently brown hens.
Distance moved (m)	The overall distance (m) moved by the hen in the arena across the duration of the test.
Mean shape	The closeness of the hen shape to a circle, where a value of 1 = circular, and a value of 0.5 indicates the ellipse is twice as long as wide.
SD shape	The standard deviation of the shape across the length of the test. A higher number means the hen changed shape more often. A hen that was moving typically had a higher SD, but stationary hens that moved their necks out to the sides also had a high SD.
Max 10-s move	The maximum distance that a hen moved in a single 10-s period. Flighty hens may score high in this category.
Stationary time (s)	The amount of time that the hen stayed still long enough to not be detected as moving. The threshold was 5-cm movement within a 10-s period.
Move ratio	The ratio of moving time to non-moving time. If a hen spent more time moving to non-moving, the ratio would be higher.

**Table 2:** Description of the measurements used in the acoustic spectrogram analysis [15,45].

Measurement	Description
Centre frequency (Hz)	The frequency dividing the fundamental frequency into two frequency intervals of equal energy.
Frequency 25% (Hz)	The frequency dividing the fundamental frequency into two frequency intervals containing 25% and 75% of the energy.
Frequency 75% (Hz)	The frequency dividing the fundamental frequency into two frequency intervals containing 75% and 25% of the energy.
Interquartile frequency	The difference between the 1st and 3rd quartile frequencies.
Frequency 5% (Hz)	The frequency dividing the fundamental frequency into two frequency intervals containing 5% and 95% of the energy.
Frequency 95% (Hz)	The frequency dividing the fundamental frequency into two frequency intervals containing 95% and 5% of the energy.
Bandwidth 90% (Hz)	The difference between the 5% and 95% frequencies.
Delta frequency (Hz)	The difference between the upper and lower frequency limits of the fundamental frequency.
Peak frequency (Hz)	The frequency at which the maximum amplitude occurs within the fundamental frequency.
Delta time (s)	The difference between the beginning time and ending time for the fundamental frequency.

**Table 3:** The duration of tonic immobility (TI), latency to first move, latency to vocalise, and latency to step in the open field test (OFT), and the latency to step out and latency to emerge in the emergence test (ET) of free-range hens from different rearing treatments and ranging patterns at 62 to 63 weeks of age (values are presented as least squares means (LSM) ± SEM). ^a,b^ Dissimilar superscript letters indicate significant differences between ranging patterns. Raw values are presented, with the analyses conducted on the transformed data.

Behaviour Test	Latency/Duration	Treatment	Category	*N*	Time/Latency (s)	F-Stats	*p*
TI	Duration	Rearing	Control	44	144.18 ± 18.84	F_2, 13_._37_ = 1.01	0.39
			Novelty	45	109.88 ± 18.56		
			Structural	46	136.34 ± 18.44		
		Ranging	Indoor	66	143.19 ± 15.35	F_2, 13_._40_ = 1.41	0.26
			Outdoor	69	117.08 ± 15.05		
OFT	Latency to move	Rearing	Control	44	10.41 ± 1.41	F_2, 10_._56_ = 0.96	0.41
			Novelty	45	9.59 ± 1.39		
			Structural	46	9.77 ± 1.36		
		Ranging	Indoor	66	11.72 ± 1.15	F_1, 10_._70_ = 1.40	0.26
			Outdoor	69	8.13 ± 1.11		
	Latency to vocalise	Rearing	Control	44	84.57 ± 15.74	F_2, 13_._56_ = 0.65	0.54
			Novelty	45	86.15 ± 15.47		
			Structural	46	75.15 ± 15.29		
		Ranging	Indoor	66	111.70 ±12.84 ^a^	F_2, 13_._63_ = 8.61	**0.01**
			Outdoor	69	52.21 ± 12.48 ^b^		
	Latency to step	Rearing	Control	31	168.45 ± 15.00	F_2, 13_._16_ = 2.75	0.10
			Novelty	30	147.10 ± 14.75		
			Structural	29	146.73 ± 14.55		
		Ranging	Indoor	31	195.80 ±12.24 ^a^	F_1, 13_._23_ = 12.65	**0.003**
			Outdoor	59	112.38 ±11.88 ^b^		
ET	Latency to step out	Rearing	Control	44	129.84 ± 17.27	F_2, 14_._19_ = 0.09	0.92
			Novelty	45	132.96 ± 16.97		
			Structural	46	138.81 ± 16.78		
		Ranging	Indoor	66	187.50 ±14.08 ^a^	F_1, 14_._28_ = 20.83	**0.0004**
			Outdoor	69	80.24 ± 13.70 ^b^		
	Latency to emerge	Rearing	Control	44	136.61 ± 17.03	F_2, 14_._13_ = 0.08	0.92
			Novelty	45	140.06 ± 16.74		
			Structural	46	142.90 ± 16.55		
		Ranging	Indoor	66	195.53 ±13.89 ^a^	F_1, 14_._23_ = 26.86	**0.0001**
			Outdoor	69	84.18 ± 13.51 ^b^		

**Table 4:** Different parameters of the automated tracking in the open field test of free-range hens from different rearing treatments and ranging patterns at 62 weeks of age (values are presented as least squares means (LSM) ± SEM).

Parameters	Rearing Treatment			F-Stats, *p*	Ranging		F-stats, *p*
	Control	Novelty	Structural		Indoor	Outdoor	
Distance moved (m)	1.43 ± 0.34 ^b^	2.98 ± 0.35 ^a^	1.76 ± 0.35 ^b^	F_2, 5_._85_ = 5.29, **0.04**	0.92 ± 0.30	3.20 ± 0.27	F_1, 4_._23_ = 84.90, **0.0006**
Mean shape	0.63 ± 0.01 ^a^	0.57 ± 0.01 ^b^	0.60 ± 0.01 ^ab^	F_2, 11_._62_ = 3.97, **0.04**	0.61 ± 0.01	0.59 ± 0.01	F_1, 11_._74_ = 2.35, 0.15
SD ^1^ of shape	0.08 ± 0.004	0.07 ± 0.004	0.07 ± 0.004	F_2, 12_._49_ = 0.76, 0.49	0.06 ± 0.003	0.09 ± 0.003	F_1, 12_._61_ = 47.07, **<0.0001**
Maximum move in 10 s	0.21 ± 0.01	0.34 ± 0.02	0.24 ± 0.02	F_2, 10_._77_ = 2.16, 0.16	0.13 ± 0.01	0.40 ± 0.02	F_1, 10_._06_ = 75.32, **<0.0001**
Stationery time (s)	245.93 ± 7.93 ^a^	201.91 ± 8.06 ^b^	231.98 ± 7.91 ^a^	F_2, 4_._75_ = 7.92, **0.03**	265.73 ± 7.63	187.48 ± 5.82	F_1, 3_._94_ = 64.79, **0.001**
Move ratio	0.44 ± 0.48	1.11 ± 0.21	0.62 ± 0.27	F_2, 13_._75_ = 0.12, 0.89	0.34 ± 0.33	1.11 ± 0.22	F_1, 12_._42_ = 10.18, **0.008**

^a,b^ Dissimilar superscript letters among values in the same row of each parameter differ significantly (*p* < 0.05). Raw values are presented, with the analyses conducted on the transformed data. Descriptions of each parameter are presented in Table 1. ^1^ Standard deviation.

**Table 5:** The number (#) of calls and number (#) of syllables per call produced by hens from different rearing treatments and ranging patterns in the open field test of free-range hens at 62 weeks of age (values are presented as least squares means (LSM) ± SEM). ^a,b^ Dissimilar superscript letters indicate significant differences between the ranging patterns. Raw values are presented, with the analyses conducted on the transformed data.

Treatment	Category	# Calls	# Syllables/Call
Rearing	Control	9.14 ± 1.13	1.58 ± 0.39
	Novelty	9.10 ± 1.20	1.87 ± 0.40
	Structural	9.64 ± 1.10	2.13 ± 0.39
F-stats, *p*		F_2, 14_._34_ = 0.23, 0.80	F_2, 13_._84_ = 0.47, 0.64
Ranging	Indoor	7.34 ± 1.02 ^b^	1.47 ± 0.34
	Outdoor	11.24 ± 0.85 ^a^	2.26 ± 0.30
F-stats, *p*		F_1, 15_._25_ = 8.69, **0.01**	F_1, 14_._42_ = 3.03, 0.10

**Table 6:** Different parameters measured from the spectrogram of the syllables produced by hens from different rearing treatments and ranging patterns in an open field test of free-range hens at 62 weeks of age (values are presented as least squares means (LSM) ± SEM).

Parameters	Rearing Treatment			F-Stats, *p*	Ranging		F-Stats, *p*
	Control	Novelty	Structural		Indoor	Outdoor	
Center Frequency (Hz)	552.13 ± 23.01	577.29 ± 24.23	572.64 ± 22.10	F_2, 14_._40_ = 0.34, 0.72	541.26 ± 20.95	593.44 ± 17.08	F_1, 15_._81_ = 3.72, 0.07
Frequency 25% (Hz)	520.64 ± 22.57	542.80 ± 23.77	537.65 ± 21.66	F_2, 13_._83_ = 0.26, 0.77	508.05 ± 20.57	559.35 ± 16.73	F_1, 15_._21_ = 3.74, 0.07
Frequency 75% (Hz)	581.05 ± 24.38	604.51 ± 25.58	604.39 ± 23.45	F_2, 15_._31_ = 0.57, 0.58	571.06 ± 22.09	622.25 ± 18.15	F_1, 16_._45_ = 3.32, 0.09
Interquartile frequency (Hz)	58.03 ± 7.28	61.02 ± 7.39	66.84 ± 7.04	F_2, 8_._99_ = 0.36, 0.70	62.61 ± 6.43	61.32 ± 5.43	F_1, 9_._71_ = 0.29, 0.60
Frequency 5% (Hz)	450.10 ± 21.98	468.44 ± 23.28	462.94 ± 21.03	F_2, 14_._00_ = 0.18, 0.84	432.73 ± 20.19	488.26 ± 16.21	F_1, 15_._56_ = 4.59, **0.04**
Frequency 95% (Hz)	621.83 ± 26.41	647.19 ± 27.62	651.84 ± 25.45	F_2, 15_._25_ = 0.60, 0.56	616.86 ± 23.82	663.71 ± 19.72	F_1, 16_._30_ = 2.52, 0.13
Bandwidth 90% (Hz)	164.89 ± 11.73	176.63 ± 12.16	183.60 ± 11.15	F_2, 9_._23_ = 0.88, 0.45	177.65 ± 10.74	172.42 ± 8.50	F_1, 11_._16_ = 0.52, 0.49
Delta frequency (Hz)	348.88 ± 19.44	357.53 ± 19.61	397.17 ± 18.92	F_2, 11_._46_ = 1.95, 0.19	371.32 ± 16.94	364.40 ± 14.71	F_1, 12_._19_ = 0.15, 0.70
Peak frequency (Hz)	558.32 ± 23.71	581.91 ± 24.79	569.48 ± 22.79	F_2, 14_._30_ = 0.24, 0.79	543.87 ± 21.45	595.93 ± 17.62	F_1, 15_._64_ = 3.51, 0.08
Delta time (s)	1.01 ± 0.07 ^ab^	0.99 ± 0.08 ^b^	1.19 ± 0.07 ^a^	F_2, 10_._66_ = 3.65, **0.06**	1.09 ± 0.07	1.04 ± 0.05	F_1, 12_._22_ = 0.88, 0.37

^a,b^ Dissimilar superscript letters within the values in the same row of each parameter differ significantly (*p* < 0.05) Raw values are presented, with the analyses conducted on the transformed data. Descriptions of each parameter are presented in Table 2.

**Table 7:** Variations in the latency to peck a novel object by the first 3 hens based on the rearing treatments and locations of the objects in the novel object test (pen level) of free-range laying hens at 63 weeks of age.

Novel Objects	Treatments	Category	Latency (s) to Peck	F-Stats	*p*
Cardboard Box	Rearing treatment	Control	65.00 ± 14.93	F_2, 6_ = 1.85	0.24
		Novelty	26.52 ± 14.93		
		Structural	49.41 ± 14.93		
	Location	1	73.67 ± 10.06 ^a^	F_2, 70_ = 15.22	**<0.0001**
		2	42.63 ± 10.06 ^b^		
		3	24.63 ± 10.06 ^c^		
Red ball	Rearing treatment	Control	175.33 ± 41.82	F_2, 6_ = 1.66	0.27
		Novelty	129.74 ± 41.82		
		Structural	248.00 ± 41.82		
	Location	1	222.96 ± 27.41 ^a^	F_2, 68_ = 9.07	**<0.0003**
		2	193.89 ± 27.41 ^a^		
		3	136.22 ± 27.41 ^b^		

^a–c^ Different superscript letters within the values of the same column of each variable differ significantly (*p* < 0.05). Raw values are presented, with the analyses conducted on the transformed data.

## Data Availability

Data will be made available upon any reasonable request to the corresponding author.
